# Curcumin attenuates α-synuclein pathology in Parkinson’s disease model mice through modulation of UBC9-associated SUMOylation signaling

**DOI:** 10.3389/fnins.2026.1885786

**Published:** 2026-07-21

**Authors:** Yao Li, Zhuang Zhu, Kezhong Zhang

**Affiliations:** Department of Neurology, The First Affiliated Hospital with Nanjing Medical University, Nanjing, China

**Keywords:** curcumin, neuroprotection, SUMOylation, UBC9, α-synuclein

## Abstract

**Background:**

Post-translational modifications, particularly SUMOylation, plays a crucial role in α-synuclein (α-syn) aggregation, a key pathological feature of Parkinson’s disease (PD). Curcumin, a natural polyphenol, has shown neuroprotective potential, but its effects on SUMOylation-related signaling in PD remain unclear.

**Objective:**

This study aimed to investigate whether curcumin modulates α-syn SUMOylation and to elucidate the underlying molecular mechanisms in PD model mice.

**Methods:**

A PD model was established in male C57BL/6 mice via unilateral intrastriatal injection of α-syn preformed fibrils (PFFs). Six months after α-syn PFFs injection, mice were treated intravenously with curcumin (25 mg/kg/day) or vehicle for 1 month. Behavioral tests (open field, rotarod) assessed motor function. Neuropathology was evaluated by immunohistochemistry and western blotting for tyrosine hydroxylase (TH), phosphorylated α-syn (p-syn), SUMOylation pathway components (SUMO1, SAE2, UBC9, PIAS1/2), and ubiquitin. Striatal dopamine levels were measured by HPLC.

**Results:**

Curcumin treatment ameliorated motor deficits and anxiety-like behaviors in PD mice. It partially preserved dopaminergic neurons and reduced p-syn aggregation in the substantia nigra, accompanied by increased striatal dopamine levels. Mechanistically, curcumin was associated with reduced SUMO1 and increased ubiquitin levels, suggesting modulation of SUMOylation-related signaling. Among SUMOylation enzymes, UBC9 expression was decreased, whereas E1 (SAE2) and E3 (PIAS1/2) components were not substantially affected.

**Conclusion:**

Our findings demonstrated that curcumin exerted neuroprotective effects in a PD model by attenuating α-syn pathology. The protective mechanism involves the inhibition of α-syn SUMOylation, primarily through the downregulation of the UBC9 enzyme. This study identifies UBC9-mediated SUMOylation as a potential target for curcumin and highlight a promising strategy for modifying α-syn-associated pathology in PD.

## Introduction

Parkinson’s disease (PD) is characterized by the gradual death of dopaminergic neurons in the substantia nigra (SN), and the accumulation of alpha-synuclein (α-syn) (Hill et al., 2023). As the second most common progressive neurodegenerative disease, PD affects over 10 million individuals globally, with a prevalence estimated to reach 13 million by 2040, thus posing a major threat to public health (Savica et al., 2016).

Current first-line PD therapies primarily rely on dopamine-replacement strategies to alleviate symptoms (Bloem et al., 2021). However, these treatments become less efficient and can result in serious complications as the disease advances (Armstrong and Okun, 2020). Therefore, finding new therapeutic targets for the development of disease-modifying treatments has emerged as a critical research frontier.

The pathogenesis of PD is increasingly linked to post-translational modifications (PTMs), which dictate the folding, stability, and toxicity of α-syn (Shahnawaz et al., 2020). Among these, SUMOylation, the covalent attachment of Small Ubiquitin-like Modifiers (SUMOs), has been highlighted as s a key but complex regulator of α-syn proteostasis (Krumova et al., 2011; Oh et al., 2011; Weetman et al., 2013). While some studies suggest that site-specific SUMOylation (predominantly at Lys96 and Lys102) can enhance α-syn solubility and inhibit its initial fibrillation (Krumova et al., 2011; Shahpasandzadeh et al., 2014), a growing body of evidence indicates that the dysregulation of the SUMOylation machinery, particularly involving the SUMO1 isoform, is intimately associated with PD pathology.

Specifically, mammals express multiple SUMO paralogs (e.g., SUMO1-5), in *α*-syn pathologies, SUMO1 modulates several pathways connected to α-syn biology and pathology progression (Dorval and Fraser, 2006; Rott et al., 2017). This functional dichotomy suggests that the impact of SUMOylation is highly context-dependent, contingent upon the specific SUMO paralogs involved and the cellular stress environment. The SUMOylation cascade requires sequential enzymatic steps involving E1 activation, E2 conjugation (exclusively mediated by UBC9 in humans), and E3 ligation (Henley et al., 2014; Vijayakumaran et al., 2015). Given its central role in modulating *α*-syn dynamics, targeting the key enzymes of the SUMOylation pathway represents a promising therapeutic strategy for α-synuclein-related pathological processes in PD.

Natural compounds, such as curcumin (a polyphenol derived from *Curcuma longa*), have demonstrated neuroprotective potential in various models of neurodegenerative diseases, including PD (Ruiz de Porras et al., 2021; Zia et al., 2021; Li et al., 2023; Cai et al., 2024). These effects are largely attributed to curcumin’s ability to mitigate oxidative stress, endoplasmic reticulum stress, and neuroinflammation (Shakeri et al., 2019; Cao et al., 2020; Cai et al., 2025). Several lines of evidence suggest that curcumin may interact with the SUMOylation pathway. For example, curcumin has been reported to modulate other PTMs, including ubiquitination and acetylation, which share enzymatic components or cross-talk with SUMOylation (Ben et al., 2011; Seo et al., 2018). In addition, direct studies have shown that curcumin treatment can alter SUMO1 conjugation and the expression of SUMO-specific proteases (SENPs) in neurological cell models (Hoppe et al., 2013; Buccarello et al., 2020). Because SUMOylation is highly sensitive to oxidative and ER stress, it is plausible that some of curcumin’s protective effects involve restoring normal SUMO homeostasis. Based on these considerations, we hypothesized that curcumin might influence α-syn SUMOylation. However, whether this occurs in PD models and the underlying molecular mechanisms remain largely unknown.

In this context, the present study was designed to investigate whether curcumin modulates α-syn SUMOylation and to explore its underlying molecular mechanisms. We systematically assessed curcumin’s potential therapeutic effects using behavioral evaluations, analysis of α-syn pathology, and examination of key components of the SUMOylation pathway.

## Materials and methods

### Materials

Curcumin (purity ≥98%, according to the manufacturer’s certificate of analysis) was obtained from Solarbio (Solarbio, Beijing, China; catalog number IC0610). Its molecular formula is C₂₁H₂₀O₆, molecular weight 368.38 g/mol. The 4′,6-diamidino-2-phenylindole (DAPI) was bought from Sigma-Aldrich (St. Louis, MO, United States). The anti-tyrosine hydroxylase (TH) antibody, anti-phosphorylated α-synuclein (Psyn) antibody, anti-ubiquitin antibody, anti-SUMO1 antibody, anti-SUMO-1 activating enzyme subunit 2 (SAE2) antibody, anti-ubiquitin carrier protein 9 (UBC9) antibody, anti-protein inhibitor of activated STAT protein (PIAS)1/2 antibody, anti-α-syn antibody, and the Alexa Fluor 488-conjugated goat anti-rabbit IgG were sourced from Abcam (Abcam, Cambridge, United Kingdom). Purification of recombinant α-syn and generation of *α*-syn pre-performed fibrils (α-syn PFFs) were conducted as described elsewhere (Volpicelli-Daley et al., 2011; Volpicelli-Daley et al., 2014).

### *In vivo* animal studies

Male C57BL/6 mice (approximately 25 g) were provided by the Laboratory Animal Centre of Sun Yat-Sen University (Guangzhou, China). The animals were housed in a standard animal facility and had *ad libitum* access to water and standard food. All the performed animal experiments conformed with the Guide for the Care and Use of Laboratory Animals and the Institutional Animal Care and Use Committee (IACUC) of the Sun Yat-Sen University approved the experiments (Approval No. SYSU-IACUC-2021-000751).

### Stereotaxic injection of α-syn preformed fibrils (PFFs)

The animal model of PD was induced by left intrastriatal injection of α-syn PFFs. Two-month-old mice were anesthetized using 10% chloral hydrate (350 μL/100 g body weight, intraperitoneal injection) and then placed on the stereotaxic frame (RWD, Guangdong, China). A midline incision was made over the skull after sterilization of the surgical site with 75% alcohol. Then, the left dorsal striatum was targeted according to the following coordinates from the bregma: anterior posterior (AP): + 0.2 mm; mediolateral (ML): −2.0 mm; dorsal ventral (DV): −2.6 mm, based on the Franklin and Paxinos mouse brain atlas (Paxinos and Franklin, 2003). A total of 5 μg (1.5 μL) of α-syn PFFs were injected at 0.27 μL/min (constant flow) using a 10 μL Hamilton syringe (Hamilton, Graubünden, Switzerland) with a 26 s gauge custom needle (Luk et al., 2012; Karampetsou et al., 2017). The needle was left in place for 5 min, before being withdrawn slowly after each injection to prevent reflux of the solution. The incision was then closed using a suture. The sham group was treated using an equal volume of normal saline (NS). The anesthetized animals were placed on an insulation blanket and monitored after injection until palinesthesia. While 10% chloral hydrate was employed for anesthesia in this study in accordance with institutional ethical approvals, we recognize that volatile anesthetics such as isoflurane offer better animal welfare. Future studies in our laboratory will prioritize the transition to isoflurane or other refined anesthetic protocols to ensure optimal animal care.

### Pole test

The pole test is a typically used to assess the agility and coordinating ability in PD model mice (Ogawa et al., 1985). The specific procedure of the pole test was adapted from the protocol described by Luchtman et al. (2009). Briefly, a gauze-wrapped rod with a length of 55 cm and a diameter of 1 cm was used as the pole and was placed vertically on the base. A wooden ball with a diameter of 3 cm was wrapped in gauze and glued on the top of pole. The mice were placed on the ball, and the times taken by each mouse to turn its nose down (primary measurement) and climb down the pole (secondary measurement) were noted. The mice received pre-training for 1 week before the formal test. Each mouse performed three successive trials with a 5-min interval during pre-modeling, post-modeling, and post-treatment sessions. The average of the three trials was determined.

### Rotarod test

The rotarod test is used to evaluate motor coordination and balance in PD model mice (Shiotsuki et al., 2010). The mice were tested using a rotarod device (YLS-4C, YiYan Science and Technology Development Co., Ltd. Shandong, China) in acceleration mode (increasing the speed from 4 rpm to 40 rpm over 90 s) based on the manufacturer’s instructions. The elapsed time when the mouse fell down from the spindle was automatically recorded by the apparatus. The mice were pre-trained on the rotarod at constant speed of 10 rpm for 30 min at least 2 d before the formal test. The performance of each mouse was measured during pre-modeling, post-modeling, and post-treatment sessions.

### Open field test

To measure the spontaneous locomotor activity and anxiety-like behaviors of mice, the open field test was conducted as described previously (Kraeuter et al., 2019). The open-field box is 40 × 40 × 40 cm with a camera directly above the arena. A mouse was gently placed into the center of the arena and the movement of the mouse was tracked by a camera for 5 min. Total distance, mean speed, distance in Zone-periphery and time in Zone-periphery were analyzed by behavioral tracking software Labmaze V3.0 system (Beijing Zhongshi Dichuang Science and technology development Co., Ltd. Beijing, China). The arena was sprayed with 70% ethanol and wiped with paper towel between each trial.

### Tissue preparation

Animals were anaesthetized using 10% chloral hydrate (350 μL/100 g body weight) and then transcardially perfused with 30 mL of saline, followed by 100 mL cold 4% PFA in 0.01 M PBS. Their brains were removed and then fixed in 4% PFA overnight. After gradient ethanol dehydration and clearing using xylene, the brains were paraffin-embedded for coronal sectioning at 5 μm.

### Immunohistochemistry

Immunohistochemical staining of the brain sections was conducted using a Vectastain Elite ABC Universal Kit (Vector Labs, Malvern, PA, United States) based on the manufacturer’s protocol. Briefly, after melting at 60 °C, deparaffinization with xylene, and hydration with gradient ethanol, the sections were subjected to heat-mediated antigen retrieval using citrate buffer (Solarbio, Beijing, China) at pH 6.0 for 30 min. Then, the sections were blocked using 2.5% normal horse serum for 15 min and then incubated with anti-TH antibodies (1:500) diluted in buffer containing 1.5% blocking serum overnight at 4 °C. Next day, the sections were rinsed and incubated with biotinylated secondary antibody for 1 h at room temperature. The immunohistochemical signal was visualized using 3,3′-diaminobenzidine (DAB) (CWBIO, Beijing, China). Immunohistochemical staining for P-syn (1,500), ubiquitin (1,800), SUMO1 (1:250), SAE2 (1,50), UBC9 (1,250) and PIAS1/2 (1,200) were performed in the same way. Images were observed under a Nikon NI-U biological microscope (Nikon, Tokyo, Japan) and quantified using Image J software (NIH, Bethesda, MD, United States).

### Immunohistochemistry analysis

For immunohistochemical quantification, images were analyzed using Image J software. Quantitative measurements were obtained from three anatomically matched sections per animal and averaged to generate a single value for each mouse. For TH analysis, TH-positive neurons within the substantia nigra pars compacta (SNpc) were manually counted under identical imaging conditions, and values were normalized to the mean value of the sham group (% of sham). For p-syn staining, the percentage of p-syn-positive area within the SNpc was quantified by calculating the ratio of immunopositive area to the total analyzed area. For SUMO1, ubiquitin, SAE2, UBC9, and PIAS1/2 staining, immunoreactivity within the defined region of interest was quantified using ImageJ under identical threshold settings, and values were normalized to the mean value of the sham group (% of sham) prior to statistical analysis.

### Immunofluorescence

Multiplex immunofluorescence staining of brain sections was implemented using a Pano Multiplex IHC Kit (PANOVUE, Hikvision, Beijing, China) incorporating Tyramide signal amplification (TSA) to visualize and amplify the expression of SAE2, UBC9, PIAS1/2 and nuclei. Sections were first melted at 60 °C, deparaffinized with xylene, and hydrated with gradient ethanol. In the first round of staining, sections were subjected to heat mediated antigen retrieval using EDTA buffer (Solarbio) at pH 8.0 for 30 min and subsequently blocked with blocking buffer (Solarbio) for 15 min before incubation with the primary antibodies. The sections were then stained with anti-SAE2 rabbit monoclonal antibody (1:500) diluted in primary antibody dilution buffer (Beyotime, Jiangsu, China) overnight at 4 °C. Next day, the sections were rinsed and incubated with an anti-rabbit/mouse horseradish peroxidase (HRP)-conjugated secondary antibody for 10 min, followed by incubation with the Alexa Fluor 570 tyramide reagent (1:500) diluted in the amplification diluent for 10 min. The sections were rinsed and finally subjected to heat-mediated antigen retrieval according to the method above. The next round of staining for UBC9 using Alexa Fluor 520 tyramide reagent and PIAS1/2 using Alexa Fluor 650 tyramide reagent were repeated. The sections were counterstained with DAPI for 5 min and mounted with Anti-fade Mounting Medium (Beyotime), and then stored in light-proof box before observation. An Olympus BX63 fluorescence microscope was used to observe the immunoreactive signals.

### Western blotting analysis

Mouse brain tissue was cut into pieces and then homogenized using a tissue homogenizer (Shanghai Jing Xin, JXFSTPRP-CL) in lysis buffer containing 1 × radioimmunoprecipitation assay (RIPA) buffer, 1 × protease inhibitor, and 1 × phosphatase inhibitor. The obtained homogenate was placed on ice, shaken for 1 h, and then centrifuged at 14,000 × *g* for 15 min at 4 °C. The supernatant was collected, and the protein concentration was determined using a bicinchoninic acid (BCA) assay. An equal amount of protein (30 μg) was run on 12% gels and then transferred to polyvinylidene fluoride (PVDF) membranes (Immobilon: Millipore, ISEQ00010). The membranes were blocked with 5% milk solution in TBS-Tween for 1 h at room temperature. Then, the membranes were incubated with primary antibodies overnight at 4 °C. Subsequently, the membranes were incubated with an appropriate HRP-conjugated secondary antibody at room temperature for 1 h. The following antibodies were used in these experiments: anti-β-actin (1:5000), anti-TH (1:1000), anti-ubiquitin (1:1000), anti-SUMO1 (1:2000), anti-SAE2 (1:1000), anti-UBC9 (1:2000), anti-PIAS1/2 (1:2000). Densitometric analysis of the immunoreactive bands was measured using Image J software.

### High-performance liquid chromatography (HPLC) analysis

The levels of dopamine and its metabolite 3,4-dihydroxyphenylacetic acid (DOPAC) in the striatum were quantified using high-performance liquid chromatography (HPLC) as previously described (Yu et al., 2017). Briefly, striatal tissue was rapidly dissected on ice and weighed. The tissue was then homogenized in 0.1 mol/L perchloric acid. Following homogenization, the samples were centrifuged at 10,000 × g for 10 min at 4 °C. The resulting supernatants were collected and analyzed for dopamine and DOPAC content by HPLC. The concentrations of each neurotransmitter were expressed as picograms per milligram (pg/mg) of wet striatal tissue weight.

### Statistical analysis

All data were analyzed using GraphPad Prism 9.0 (GraphPad Software, San Diego, CA, United States) and SPSS 26.0 (IBM Corp., Armonk, NY, United States). Given the small group size (*n* = 6 per group) and the fact that not all datasets met the assumptions of normality (Shapiro–Wilk test) and homogeneity of variance (Levene’s test), nonparametric tests were employed throughout the study. For two-group comparisons (e.g., sham vs. α-syn, or α-syn + vehicle vs. α-syn + curcumin), the Mann–Whitney *U* test was used. For multiple-group comparisons (e.g., sham, α-syn, α-syn + vehicle, α-syn + curcumin), the Kruskal–Wallis test was applied, followed by Dunn’s *post hoc* test with Bonferroni correction for multiple comparisons.

Data are presented as mean ± SEM in the figures for consistency with previous literature, but all statistical analyses were performed on the raw ranks. *p*-value < 0.05 was considered statistically significant.

## Results

### Determination of the establishment of the PD mice model

To establish a pathologically relevant mouse model of PD, 2-month-old male C57BL/6 mice received unilateral intrastriatal injections of α-syn PFFs. The use of α-syn PFFs provides a more robust model for recapitulating the slowly progressive nature of PD, thereby facilitating a more accurate investigation into the underlying pathological mechanisms of α-syn. Transmission electron microscopy (TEM) micrograph of the α-syn PFFs is provided in Supplementary Figure 1. We first sought to validate the successful establishment of the model by evaluating dopaminergic neurodegeneration and behavioral impairments at 1, 3, and 6 months post-injection. Sham-operated mice served as the control group. Immunostaining and pole test confirmed that significant PD-like pathology and motor deficits were consistently observed at the 6-month mark (Supplementary Figure 2). Consequently, this 6-month time point was selected as the baseline for subsequent therapeutic interventions.

### Curcumin treatment protected against α-syn-induced motor dysfunction and anxiety-like behaviors

**Figure 1:**
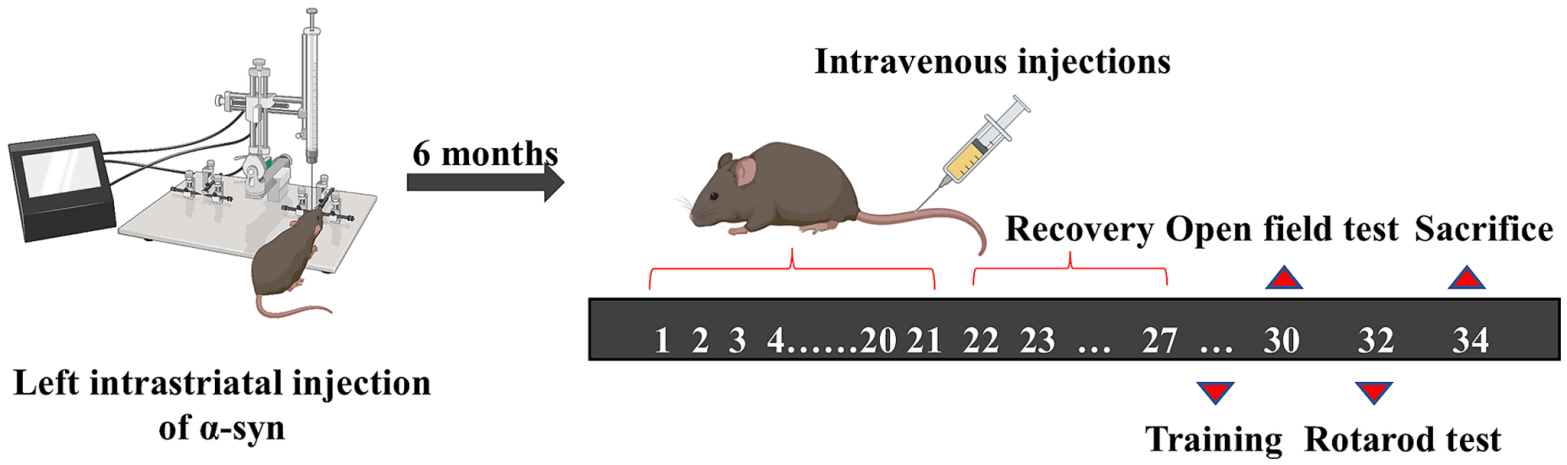
Time course of the model establishment (left intrastriatal injection of 5 μg of α-syn PFFs for 6 months), drug administration (intravenous injection of curcumin solution or DMSO dissolved in saline) and behavioral tests in experiment mice. (Some drawing materials were provided by Biorender.com).

Having established the α-syn PFFs-induced PD mice, we next investigated the neuroprotective potential of curcumin against α-syn-induced neurobehavioral deficits. Figure 1 presents a schematic of experimental time course in PD mice model. After model establishment, mice were randomly assigned into four groups: sham + vehicle group, α-syn group, α-syn + vehicle group, α-syn + curcumin group. Curcumin was administered intravenously via the tail vein at a dose of 25 mg/kg/day for 1 month based on previous experience and similar studies. Vehicle-treated mice received an identical formulation without curcumin.

Curcumin was first dissolved in 100% dimethyl sulfoxide (DMSO) to prepare a stock solution (43.75 mg/mL). Immediately before administration, the stock solution was diluted 10-fold with sterile saline to obtain a working solution containing 4.375 mg/mL curcumin in 10% (v/v) DMSO in sterile saline. The working solution was freshly prepared immediately before use, vortexed thoroughly until no visible precipitation was observed, and protected from light throughout preparation and administration. Vehicle-treated mice received an identical formulation consisting of 10% (v/v) DMSO in sterile saline without curcumin. Each mouse (approximately 35 g) received a daily intravenous tail vein injection of 200 μL, corresponding to a curcumin dose of 25 mg/kg (0.875 mg per mouse). All injections were administered slowly over 30–60 s to minimize injection-related adverse effects.

Following completion of the treatment period, mice were monitored for an additional 2 weeks during which a series of behavioral tests was performed. Animals were sacrificed on the final experimental day for subsequent biochemical and histological analyses. To assess motor coordination and emotional status, mice underwent the open field test (OFT).

**Figure 2:**
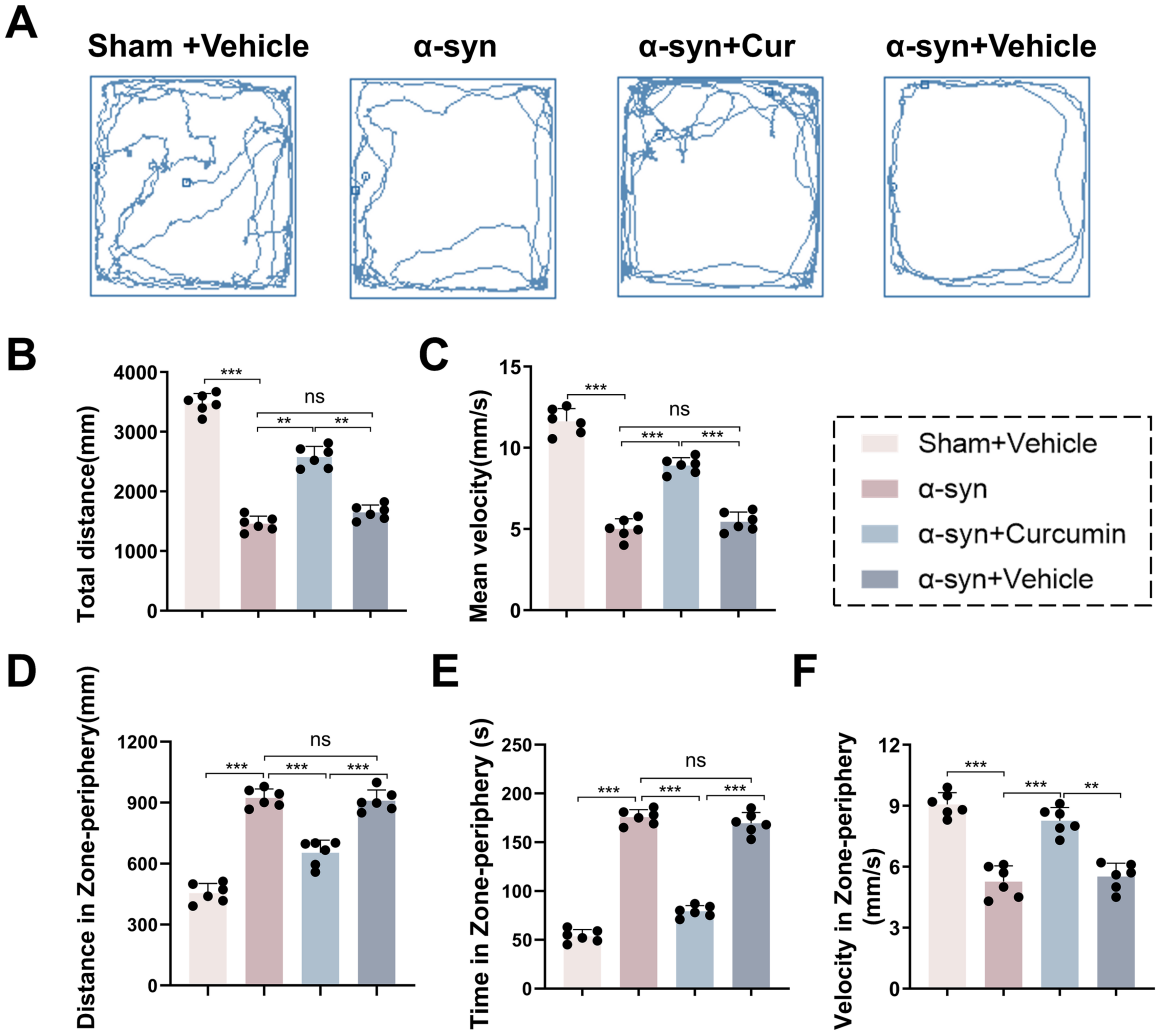
Protective effect of intravenous administration of curcumin on the α-syn mouse model of PD. **(A)** Open field test. Representative movement traces (blue) and parameters analysis showing changes in **(B)** total distance and **(C)** mean speed of different groups of mice. Changes in anxiety status of treated mice were assessed by the **(D)** distance in zone-periphery, **(E)** time in zone-periphery and **(F)** speed in zone-periphery in the open field test. Data are shown as the mean ± SEM (*n* = 6), *p < 0.05, **p < 0.01, ***p < 0.001, ****p < 0.0001.

As illustrated in Figures 2A–C, mice treated with curcumin traveled a longer distance, moved with greater average velocity around the arena than the mice that received DMSO. This result indicated that the curcumin treatment ameliorated the locomotor activity and damaged motor coordination of the mice induced by *α*-syn. Furthermore, the OFT revealed that curcumin treatment effectively alleviated anxiety-like behaviors; these mice spent significantly less time in the periphery and exhibited increased exploratory activity compared to the vehicle-treated PD group (Figures 2D–F). In contrast, the vehicle (DMSO) alone showed no therapeutic effect on either motor or emotional indices. In addition to the improved locomotor activity observed in the OFT, the rotarod test further demonstrated that curcumin enhanced motor coordination in the *α*-syn PFFs-induced PD mice (Supplementary Figure 3).

### Curcumin attenuated dopaminergic neuronal loss and α-syn pathology

To determine whether the observed behavioral improvements correlated with neuroprotection in the brain, we examined the survival of dopaminergic neurons in the substantia nigra pars compacta (SNpc). IHC and Western blot analysis of tyrosine hydroxylase (TH) were performed to quantify neuronal integrity.

**Figure 3:**
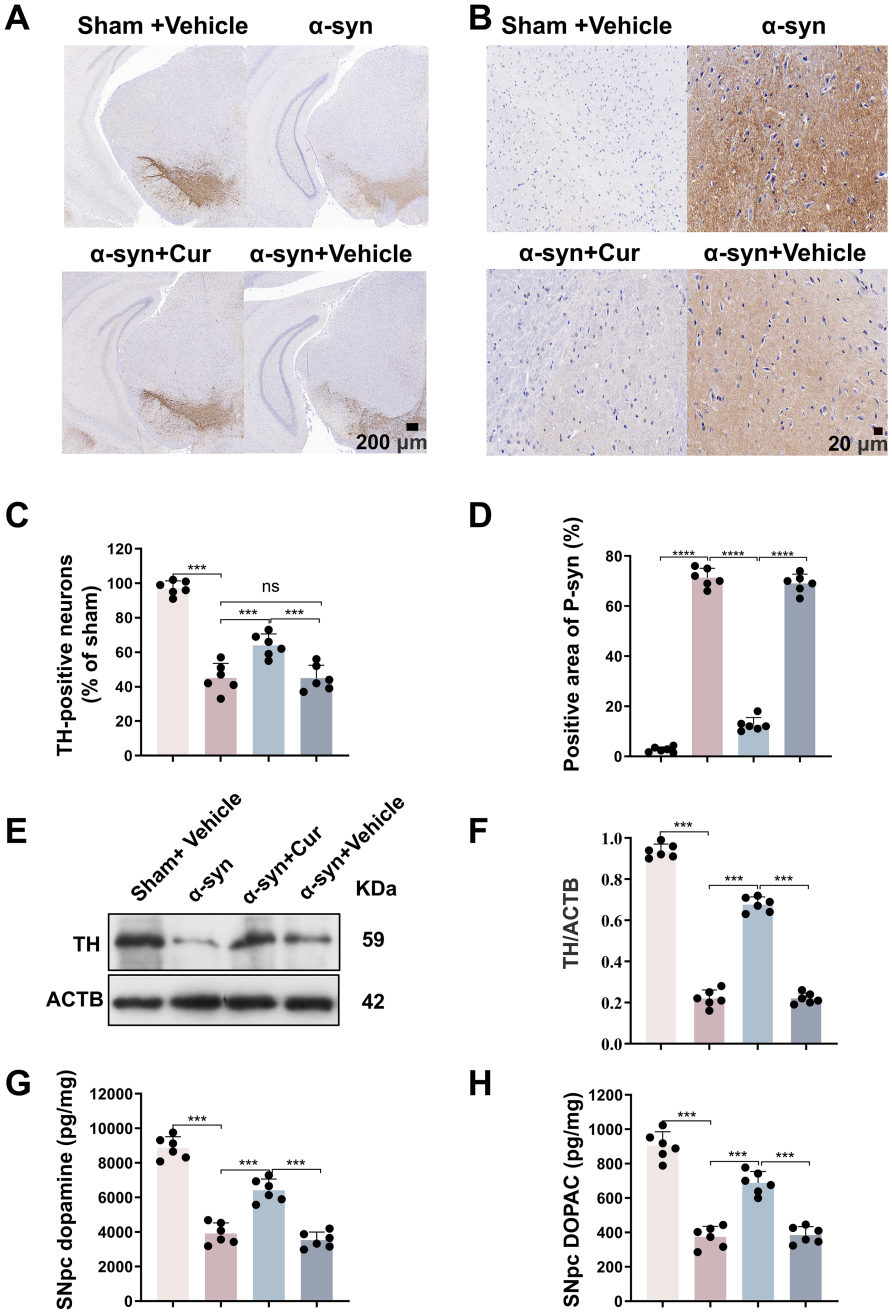
Protective effect of intravenous administration of curcumin against dopaminergic system degeneration and α-syn accumulation in the PD model mice. **(A)** TH immunostaining of midbrain sections excised from the different treatment groups. **(B)** Representative images of the accumulation of phosphorylated α-syn (P-syn) in the left SN of different groups of mice. **(C)** Quantification of TH-positive neurons in the SNpc, expressed as percentage of the sham group mean (% of sham). **(D)** Quantification of p-syn-positive area in the SNpc, expressed as percentage of the total analyzed area (%). **(E)** Representative blots showing TH expression in the left SN of different groups. Relative intensity was normalized to that of **(F)** TH/ACTB. SN dopamine **(G)** and its metabolites DOPAC levels **(H)** measured by HPLC. Data are shown as the mean ± SEM (*n* = 6), *p < 0.05, **p < 0.01, ***p < 0.001, ****p < 0.0001.

Immunostaining results showed that curcumin treatment preserved TH immunoreactivity and reduced pathological phosphorylated α-syn (P-syn) accumulation in the SNpc of PD mice (Figures 3A–D). In contrast, decreased TH levels and dense P-syn aggregation were observed in the α-syn + vehicle group. This was further supported by Western blot data, which showed a significant rescue of TH protein levels in the curcumin-treated group compared to the marked reduction seen in the α-syn + vehicle group (Figures 3E,F). These results suggested that curcumin treatment partially attenuated the loss of TH-positive neurons and the reduction in dopamine levels, although these parameters did not fully return to control levels. Besides, although the representative image for the α-syn + vehicle group shows somewhat higher p-syn immunoreactivity than that for the α-syn group, quantitative analysis across three sections per animal revealed no significant difference between these two groups.

To further assess dopaminergic functional status, dopamine (DA) and its metabolite DOPAC were measured by HPLC. Curcumin-treated mice exhibited significantly higher DA and DOPAC levels in the SN compared with the α-syn group (Figures 3G,H). Together with the behavioral and histopathological findings, these results suggest that curcumin partially ameliorated α-syn-associated dopaminergic dysfunction in this model. However, because DA/DOPAC measurements and TH expression alone cannot fully establish neuronal survival or disease modification, the present findings should be interpreted cautiously and within the context of *α*-syn-related pathological changes.

### Curcumin modulated the balance between SUMOylation and ubiquitination in the SNpc

To explore the molecular mechanism underlying curcumin’s neuroprotective effects, we focused on the post-translational modification of α-syn. Given that SUMOylation and ubiquitination often act antagonistically to regulate protein stability, we hypothesized that curcumin might facilitate α-syn clearance by inhibiting its SUMOylation.

**Figure 4:**
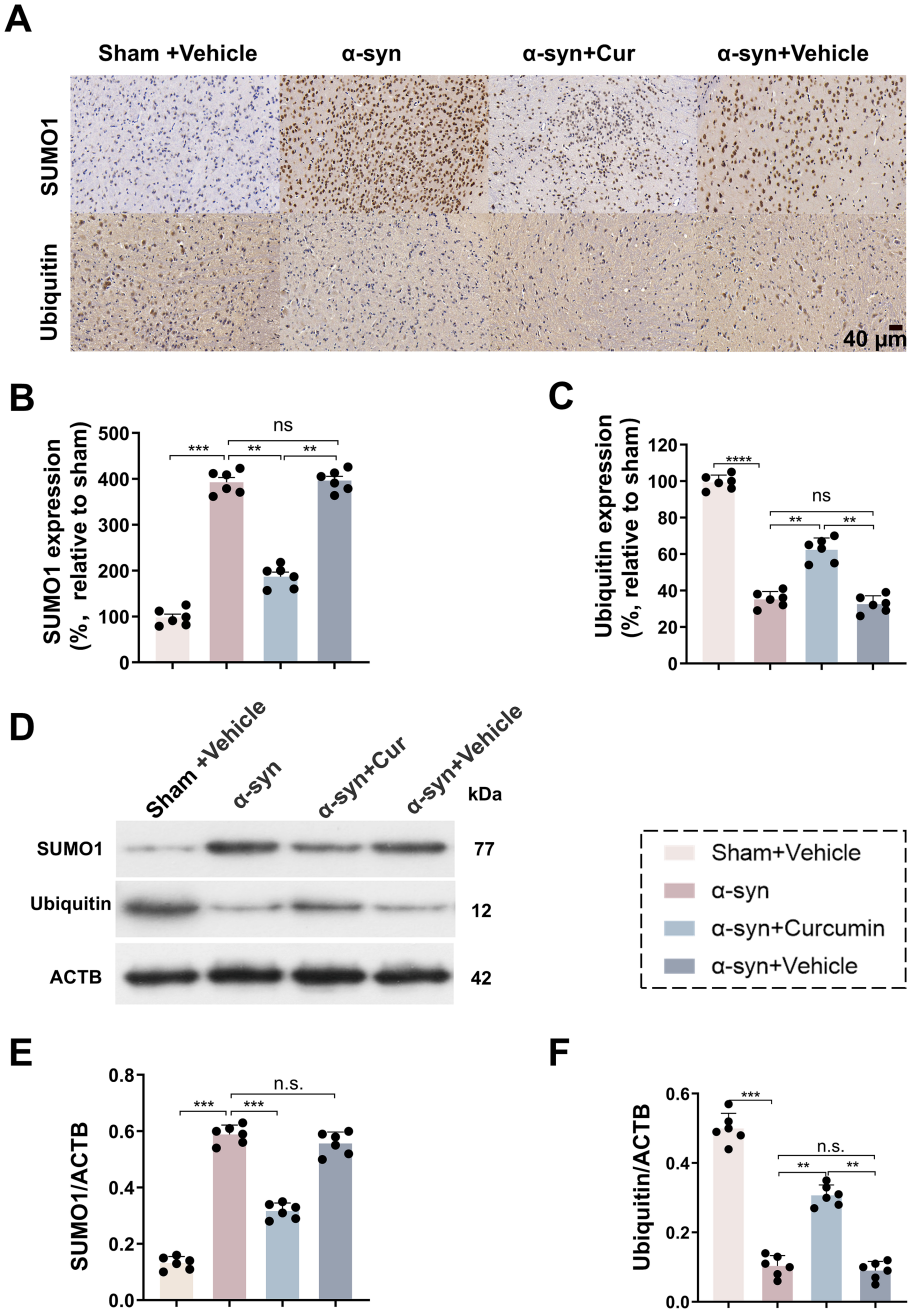
Neuroprotective effect of curcumin against accumulation of α-syn in the PD model mice by inhibiting α-syn SUMOylation. **(A)** Representative images of midbrain sections excised from different groups shown above immunstained for SUMO1 and ubiquitin. Quantification of **(B)** SUMO1 and **(C)** ubiquitin immunoreactivity in the SNpc, normalized to the mean value of the sham group (% of sham). **(D)** Representative blots showing SUMO1 and ubiquitin expression in the SN of different treatment groups. Relative intensity was normalized to that of **(E)** SUMO1 /ACTB or **(F)** ubiquitin /ACTB. Values are shown as the mean ± SEM, *n* = 6, **p < 0.01, ***p < 0.001, ****p < 0.0001.

Our analysis showed that α-syn PFFs injection led to a robust increase in SUMO1 expression and a concomitant decrease in ubiquitin expression in the SNpc, suggesting impaired protein degradation (Figures 4A–C). Remarkably, curcumin treatment significantly reversed this phenotype, decreasing SUMO1 levels while restoring ubiquitin expression (Figures 4D–F). These results, confirmed by both immunostaining and Western blotting, indicate that curcumin shifts the modification profile of α-syn away from SUMOylation and toward ubiquitination, thereby promoting its degradation.

### Curcumin reduced α-syn pathology through suppression of UBC9-associated SUMOylation signaling

Finally, we sought to identify the specific component of the SUMOylation machinery targeted by curcumin. The SUMOylation cascade involves E1 (activating), E2 (conjugating), and E3 (ligase) enzymes. We screened the expression levels of SAE2 (E1), UBC9 (E2), and PIAS1/2 (E3) in the SNpc.

**Figure 5:**
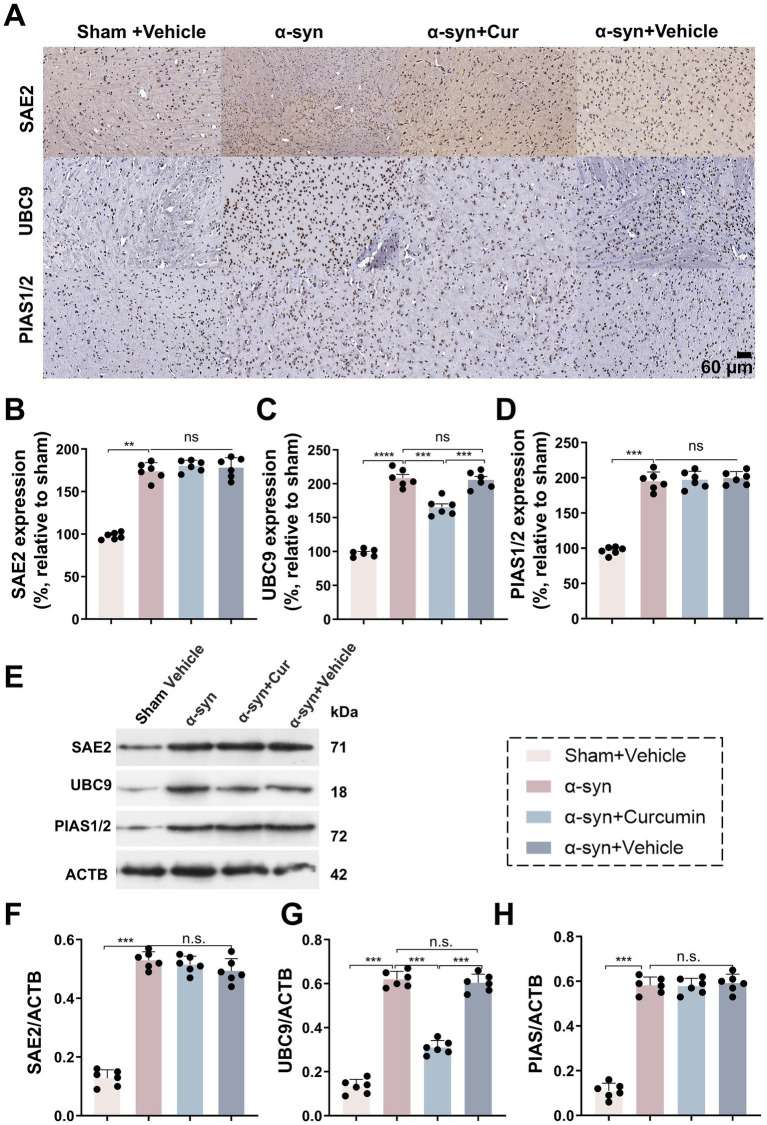
The enzymes in the SUMOylation cascade that were affected by the curcumin. **(A)** Representative images of midbrain sections excised from different groups immunstained for SAE2, UBC9 and PIAS1/2. Quantification of **(B)** SAE2, **(C)** UBC9 and **(D)** PIAS1/2 immunoreactivity in the SNpc, normalized to the mean value of the sham group (% of sham). **(E)** Representative blots showing SAE2, UBC9 and PIAS1/2 expression in the SN of different treatment groups. Relative intensity was normalized to that of **(F)** SAE2/ACTB, **(G)** UBC9/ACTB or **(H)** PIAS1/2/ACTB. Values are shown as the mean ± SEM, *n* = 6, **p < 0.01, ***p < 0.001, ****p < 0.0001.

**Figure 6:**
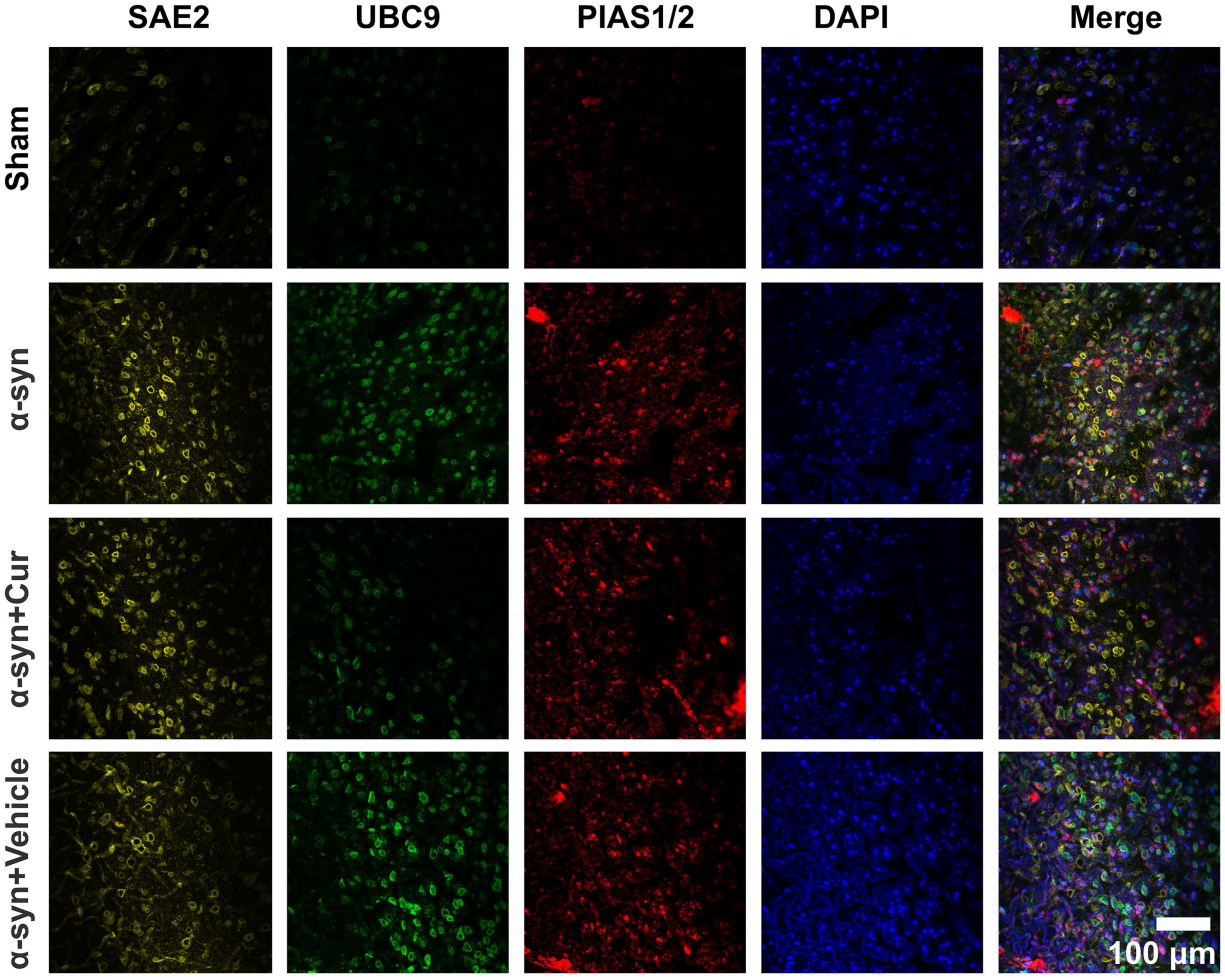
Representative multiplex immunofluorescence staining for the enzymes in the α-syn SUMOylation in the left SN of different groups of mice.

Immunostaining showed a specific and significant reduction in UBC9 expression following curcumin treatment (Figures 5A–D). Interestingly, curcumin had no significant inhibitory effect on the expression of the E1 enzyme (SAE2) or E3 ligases (PIAS1/2). These findings were further validated by Western blot analysis, which confirmed that curcumin selectively suppresses the E2 conjugating enzyme UBC9 without altering other key enzymes in the cascade (Figures 5E–H). Such curcumin modulation was also verified by multiplex immunofluorescence analysis (Figure 6). Collectively, these results demonstrate that curcumin inhibits α-syn SUMOylation primarily through the downregulation of UBC9, which in turn mitigates α-syn-mediated neurodegeneration.

## Discussion

Curcumin, a polyphenolic compound derived from *Curcuma longa*, has garnered considerable attention for its therapeutic potential in neurodegenerative disorders (Kalaycıoğlu et al., 2017; Djawad et al., 2023). Accumulating evidence supports its beneficial effects in PD models (Sharma and Nehru, 2018; Cao et al., 2020; Fikry et al., 2022; Rathore et al., 2023). In the present study, we demonstrated that curcumin exerted neuroprotective effects in α-syn PFFs-induced PD mice by attenuating α-syn pathology through suppression of UBC9-associated SUMOylation signaling. Curcumin treatment improved behavioral deficits, reduced *α*-syn aggregation, partially restored dopaminergic markers, and increased ubiquitin expression in the SNpc. In addition, curcumin administration via tail vein injection exhibited a favorable safety profile without significant alterations in transaminase levels, serum creatinine, or body weight (Supplementary Figures 4, 5).

The PD is characterized by motor symptoms including bradykinesia, resting tremor, and postural instability, as well as non-motor symptoms such as anxiety and depression (Ascherio and Schwarzschild, 2016; Faustino et al., 2020; Huang et al., 2022). In this study, behavioral experiments showed that curcumin significantly alleviated core PD manifestations in the murine model, including: improved locomotor activity; restored motor coordination; attenuated anxiety-like behaviors. These observations were consistent with protective effects of curcumin’s antioxidant and anti-inflammatory properties on dopaminergic neurons reported in previous studies (Alizadeh and Kheirouri, 2019; Lagoa et al., 2025). It is worth noting that the use of thigmotaxis in the open field test as a measure of anxiety-like behavior remains debatable. Nevertheless, several studies have demonstrated that thigmotaxis is a well-validated index of anxiety in both animals and humans, with cross-species validity (Walz et al., 2016). Recently published studies continue to employ thigmotaxis in the open field test as a reliable measure of anxiety-like behavior, further supporting its utility (Shen et al., 2025; Wang et al., 2025).

Aggregation of pathological α-syn is a central event in PD pathogenesis (Goedert, 2015; Matar and Halliday, 2025). The latest research suggests that exposure to exogenous *α*-syn, the native intrinsically disordered structure of physiological α-syn undergoes conformational transition into PFFs with demonstrated seeding potency (Bliederhaeuser et al., 2016; Longhena et al., 2017; Vargas et al., 2019). Moreover, stereotaxic intrastriatal injection of α-syn PFFs in animals models induces progressive formation of pathological α-syn inclusions not only in striatal projection neurons but also in the SNpc and piriform cortex (Pan et al., 2022; Wang et al., 2024). Based on these, we established α-syn PFFs-induced PD mice via stereotaxic intrastriatal injection of α-syn PFFs and aggregation of α-syn was obviously observed in PD mice. In our study, curcumin treatment reduced *α*-syn aggregation and partially restored TH and DOPAC levels in the SNpc of PD mice, consistent with previous studies (Sharma and Nehru, 2018; Liu et al., 2023). Together with the behavioral and histopathological findings, these results suggest that curcumin may partially ameliorate *α*-syn-associated dopaminergic dysfunction in this model.

Recent evidence suggests that PTMs critically regulate α-syn aggregation and toxicity (Kametani et al., 2024; Park et al., 2025). Among these PTMs, SUMOylation has emerged as an important modulator of α-syn biology (Sarge and Park-Sarge, 2009; Yau et al., 2020; Hassanzadeh et al., 2024). However, the role of SUMOylation in α-syn pathology is complex and appears to be context-dependent, as noted by recent critiques. Previous *in vitro* studies have suggested that direct SUMOylation of α-syn can enhance its solubility and inhibit the initial formation of fibrils (Krumova et al., 2011; Shahpasandzadeh et al., 2014). In contrast, our results, together with a growing body of *in vivo* evidence, indicated that dysregulated SUMOylation, particularly SUMO1-associated signaling, may promote α-syn accumulation (Oh et al., 2011; Rott et al., 2017). This discrepancy is primarily attributed to differences in SUMO modification sites, SUMO paralog usage, modification stoichiometry, and experimental systems (*in vitro* vs. *in vivo*).

SUMOylation appears to influence α-syn pathology in an isoform- and context-dependent manner. Previous studies suggest that α-syn SUMOylation is predominantly associated with the SUMO1 paralog, which regulates pathways relevant to α-syn aggregation and toxicity (Rott et al., 2017). In addition, pathogenic factors such as oxidative stress, inflammation, and neurotoxicity can enhance SUMO1-associated signaling and thereby promote α-syn accumulation (Ariga et al., 2013; Savyon and Engelender, 2020). Consistently, intracerebral rotenone injection in mice significantly elevates both SUMO1 and α-syn levels (Weetman et al., 2013). In the present study, curcumin treatment reduced SUMO1 expression in the SNpc of α-syn PFF-induced mice, accompanied by increased ubiquitin expression and reduced α-syn aggregation. These findings are consistent with the observation by Hirohama et al. (2013) that spectinomycin B1 inhibits *α*-syn aggregation by suppressing SUMOylation, further supporting the modulation of this PTM as a potential therapeutic intervention.

Mechanistically, protein SUMOylation consists of sequential coordinated actions of three enzymes, involving E1 SUMO activation (SAE1, SAE2), E2 SUMO-specific conjugation (UBC9, the sole E2 conjugating enzyme) and in most cases, the activity of E3 SUMO-ligase (PIAS1/2, PIAS3, hPc2, etc.), each one uniquely contributes to the completion of this cascade and the final SUMOylation of the substrate (Droescher et al., 2013; Mandel and Agarwal, 2022). Emerging evidence suggested that among several E1 SUMO activation and E3 SUMO-ligase enzymes, SAE2 and PIAS1/2 mainly play a role in PD pathogenesis (Savyon and Engelender, 2020). Our findings identified UBC9 as a potential regulator involved in the anti-SUMOylation effects of curcumin. Consistent with our findings, siRNA-mediated UBC9 knockdown has been shown to reduce pathogenic fibril aggregation (Khodzhigorova et al., 2012). Other SUMOylation inhibitors, such as ginkgolic acid, have similarly been reported to suppress SUMOylation signaling through inhibition of the E1-SUMO intermediate (Fukuda et al., 2009; Vijayakumaran et al., 2019), while inhibition of PIAS1/2 also reduces *α*-syn SUMOylation (Rott et al., 2017). Together, these observations support the concept that modulation of SUMOylation-related enzymes may represent a promising strategy for regulating *α*-syn-associated pathology.

Since SUMOylation and ubiquitination may compete for the same lysine residues (Rott et al., 2017), the curcumin-induced reduction in SUMOylation may facilitate proteasomal degradation of α-syn by shifting the PTM balance toward ubiquitination. This mechanism may partly explain the enhanced α-syn clearance observed in our study. However, the present study did not directly determine the SUMOylation or ubiquitination status of α-syn itself. The observed changes in SUMO1 and ubiquitin were measured at the total protein level and therefore may also reflect modifications of proteins other than α-syn. Consequently, although our findings support an association between curcumin treatment and altered SUMOylation-related signaling, direct evidence linking curcumin to α-syn-specific SUMOylation remains lacking. Future co-immunoprecipitation, proteasomal flux assays, and site-specific analyses will be required to further validate this mechanism. In addition, mass spectrometry and site-directed mutagenesis studies may help identify the specific α-syn SUMOylation sites regulated by curcumin.

Despite decades of clinical development, current PD therapies have not succeeded in modifying disease progression. This failure is underscored by recent clinical trial setbacks involving α-synuclein-targeting biologics and mitochondrial protectants (Testai et al., 2021; Lang et al., 2022). Our findings suggested that curcumin-mediated suppression of UBC9-dependent SUMOylation may represent a potential therapeutic approach targeting α-syn-dependent pathological mechanisms through modulating PTM dysregulation and promoting α-syn clearance.

Several limitations of the present study should also be acknowledged. First, although curcumin treatment was associated with partial restoration of TH expression and DA/DOPAC levels, these measures alone cannot definitively establish neuronal survival or disease modification and should therefore be interpreted together with the behavioral and histopathological findings. In addition, the α-syn PFF-induced model primarily reflects α-synuclein-dependent pathological processes and does not fully recapitulate the multifactorial etiology of idiopathic PD. Therefore, the present findings should be interpreted within the context of α-syn-related pathology, and future studies using complementary PD models are warranted.

Several additional mechanistic and methodological limitations should also be considered. Western blot analysis of *α*-syn solubility and aggregation states was not included in the present study. Although P-syn immunohistochemistry provided spatial evidence of reduced aggregation following curcumin treatment, future studies incorporating biochemical fractionation and aggregation-state analysis of α-syn species will be important to further validate these findings. Moreover, although changes in SUMO1 and ubiquitin expression were observed, the SUMOylation and ubiquitination status of α-syn itself was not directly determined. Therefore, the proposed mechanism should be interpreted as an association rather than definitive evidence of α-syn-specific SUMOylation. Future co-immunoprecipitation, proteasomal flux assays, mass spectrometry, and site-specific mutagenesis studies will be important to establish the direct molecular interaction between curcumin and α-syn SUMOylation. In addition, only DA and DOPAC were quantified by HPLC, whereas homovanillic acid (HVA), the final metabolite of dopamine degradation, was not measured. Consequently, dopamine turnover (e.g., HVA/DA ratio) could not be evaluated. Future studies incorporating comprehensive catecholamine metabolite profiling will provide a more complete assessment of dopaminergic metabolism. Furthermore, whether the one-month treatment paradigm confers durable neuroprotection remains unknown and should be examined in future studies employing longer observation periods and delayed-start designs.

Another important limitation relates to the formulation and delivery of curcumin. Although the curcumin formulation used in the present study produced reproducible biological effects, its physicochemical stability and pharmacokinetic properties were not formally characterized. It should also be noted that DMSO was used as the vehicle for curcumin administration. Although vehicle-treated animals received the same DMSO formulation as the curcumin-treated group, DMSO itself has reported antioxidant and neuroprotective properties and therefore cannot be regarded as a completely inert vehicle. Future studies employing alternative formulations or dedicated drug delivery systems will be important to further exclude potential vehicle-related effects. In addition, although the curcumin dose and administration route were selected based on previous literature, plasma and brain concentrations of curcumin were not measured in the present study. Consequently, the exact brain exposure levels achieved remain uncertain. Furthermore, only a single dose was evaluated, and dose-dependent effects of curcumin on SUMOylation and α-syn pathology were not investigated. Future studies employing optimized drug delivery systems, such as nanoparticle- or micelle-based formulations, together with comprehensive pharmacokinetic analyses (including HPLC-MS/MS quantification of curcumin in brain tissue) and systematic dose–response experiments, will be important to optimize the therapeutic window, minimize potential vehicle-related effects, and better define the relationship between drug exposure and therapeutic efficacy.

Finally, certain aspects of the experimental design also warrant consideration. One of limitations of this study is the absence of a Sham + Curcumin control group. Although our primary objective was to evaluate the therapeutic effects of curcumin in α-syn PFFs-induced PD mice, inclusion of this group would help distinguish disease-specific effects from the intrinsic biological actions of curcumin under physiological conditions. Future studies incorporating this control group will further strengthen interpretation of the molecular and behavioral findings. In addition, the present study was conducted exclusively in male mice. Male animals are commonly used in initial neuroprotection studies to reduce potential variability associated with hormonal fluctuations. However, given the recognized sex-specific differences in PD prevalence, symptom progression, and therapeutic responses, future studies including female cohorts will be essential to determine whether the mechanisms identified here are sex-dependent or broadly applicable.

In conclusion, our study suggested that curcumin may exert neuroprotective effects in α-syn PFFs-induced PD mice by reducing α-syn aggregation through suppression of UBC9-associated SUMOylation signaling. These findings supported a potential role for SUMOylation modulation in α-syn-related pathological processes, although the precise molecular mechanisms and long-term durability of the observed effects require further investigation.

## Data Availability

The datasets presented in this study can be found in online repositories. The names of the repository/repositories and accession number(s) can be found in the article/Supplementary material.
