# Supplementary material for: Curcumin attenuates α-synuclein pathology in Parkinson’s disease model mice through modulation of UBC9-associated SUMOylation signaling
Source: Front Neurosci. 2026 Jul 21;20:1885786. doi: 10.3389/fnins.2026.1885786 (PMC13433455; doi:10.3389/fnins.2026.1885786)
Supplement: Supplementary file 1 [file Data_Sheet_1.PDF]

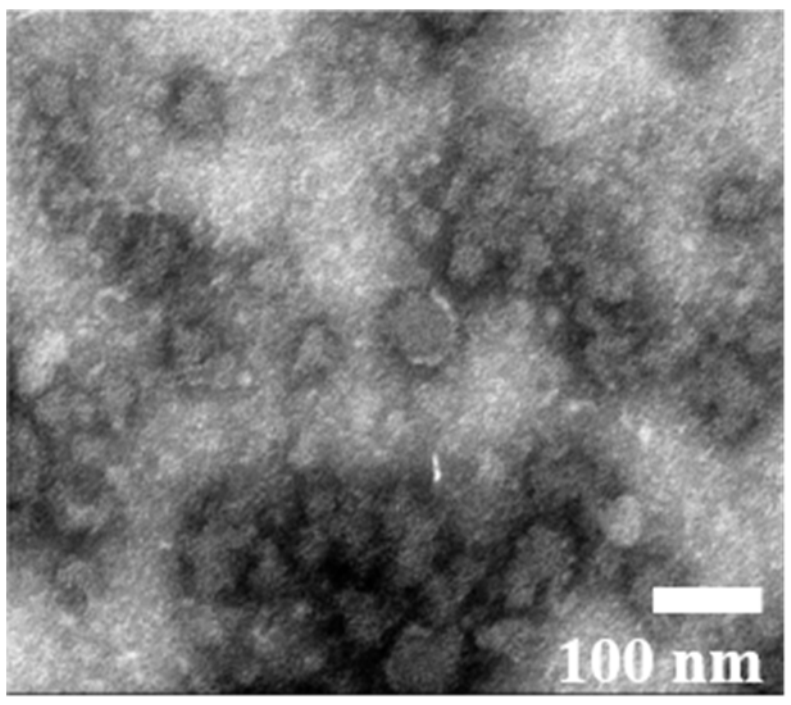

Supplementary Figure 1 A representative transmission electron microscopy (TEM) image of the α-syn PFFs used in this study.

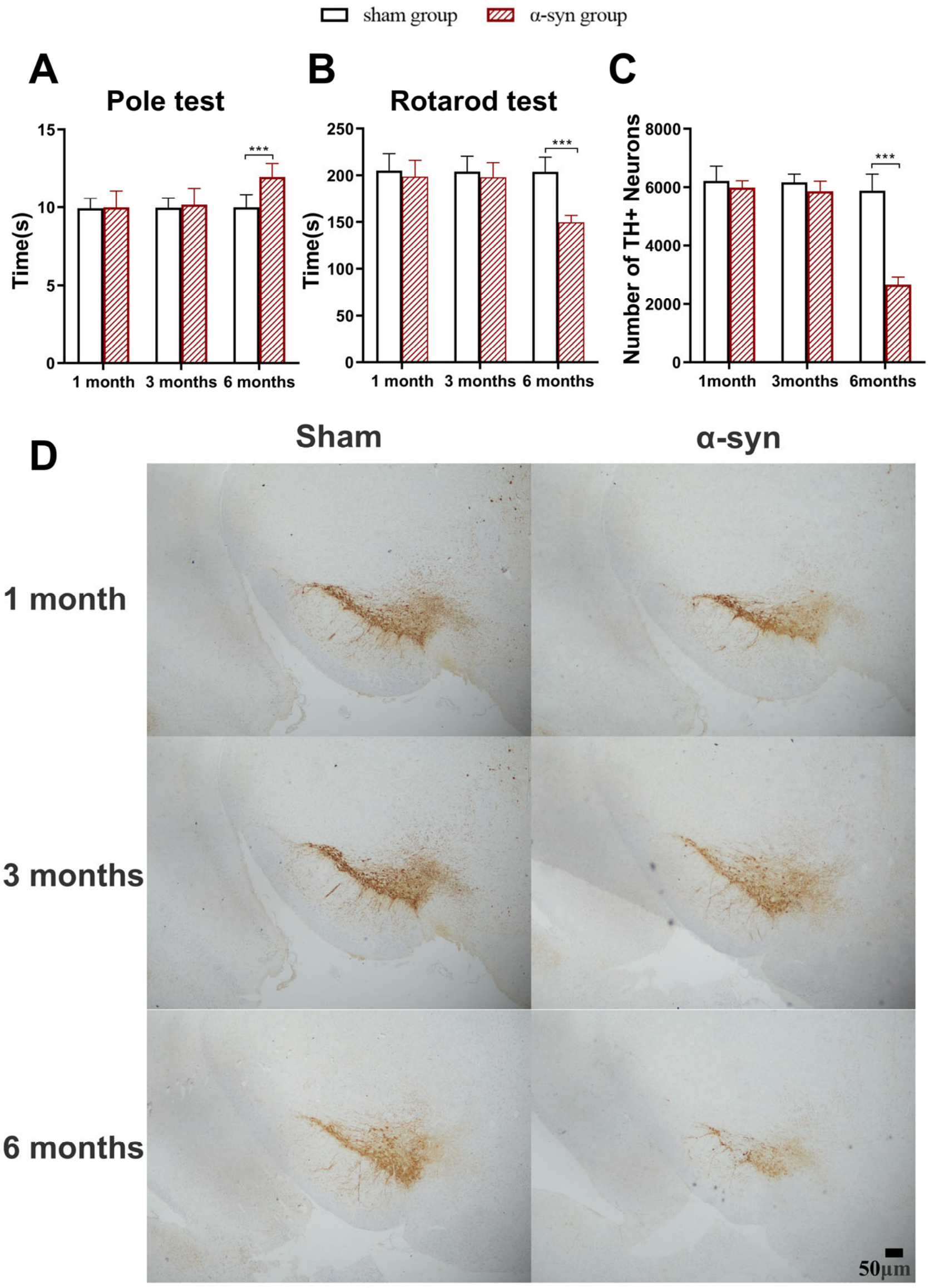

Supplementary Figure 2. At 1, 3, and 6 months after left intrastriatal injection of α-syn oligomers, behavioral experiments and TH immunostaining were used to evaluate the successful establishment of PD model mice. A) Pole test and B) rotarod test showing significant bradykinesia and uncoordinated motion in mice at 6 months after left intrastriatal injection of α-syn oligomers. C-D) TH immunostaining showing significant decreased TH expression in the left SN at 6 months after α-syn injection. Data represent mean ± SEM (n=6), ****p*<0.001. Abbreviations: TH, Tyrosine hydroxylase. SN, Substantia Nigra.

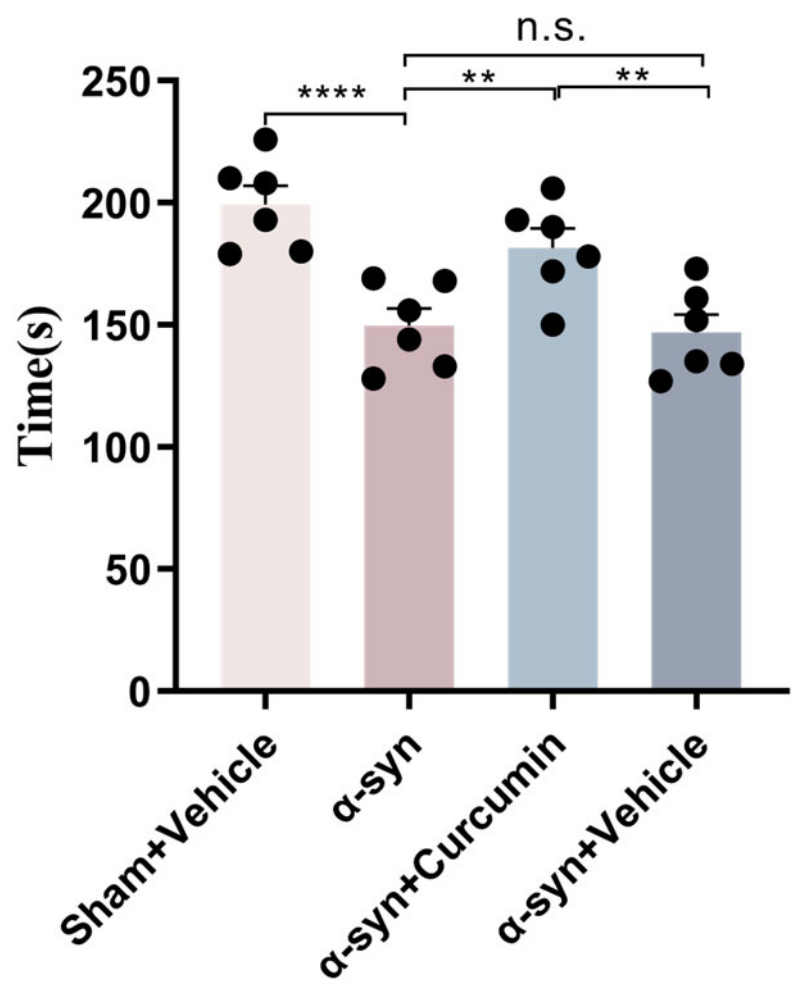

Supplementary Figure 3. **Supplementary behavioral experiments on the neuroprotective effects of curcumin in PD mice.** Changes in motor coordination and balance of treated mice were measured by the rotarod test. Mice treated with curcumin remained on the rotating rod for longer than the mice that received the vehicle. Data are shown as the mean ± SEM (n=6), **p* < 0.05, ***p*<0.01, ****p*<0.001, *****p*<0.0001.

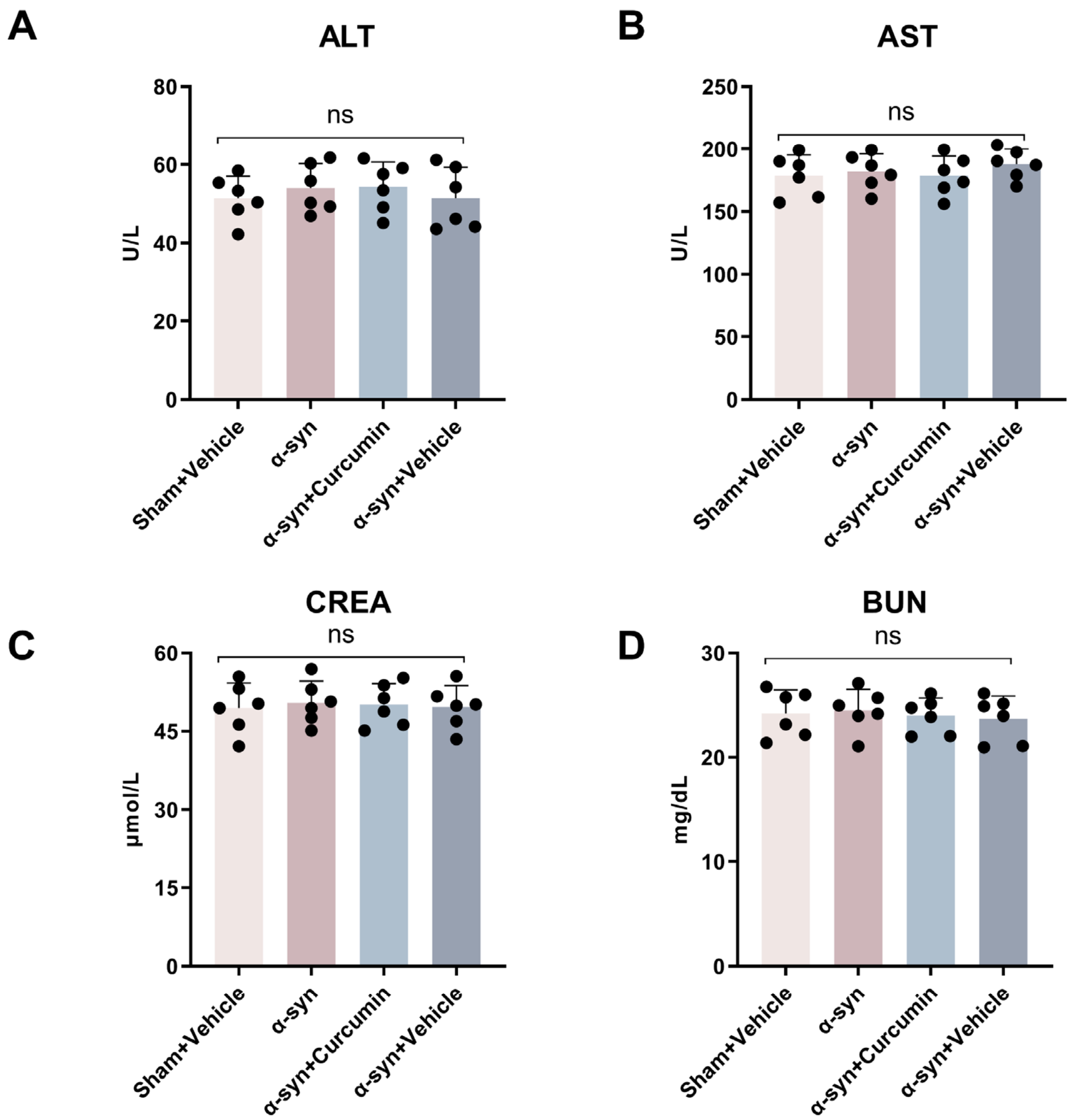

Supplementary Figure 4. **Assessment of common liver and kidney function indices of mice in all groups.** Liver and kidney functions in mice were assessed by measuring serum levels of alanine aminotransferase (ALT), aspartate aminotransferase (AST), creatinine (CREA), and blood urea nitrogen (BUN). Data are shown as the mean ± SEM (n=6), *$p < 0.05$, **$p<0.01$, ***$p<0.001$, ****$p<0.0001$.

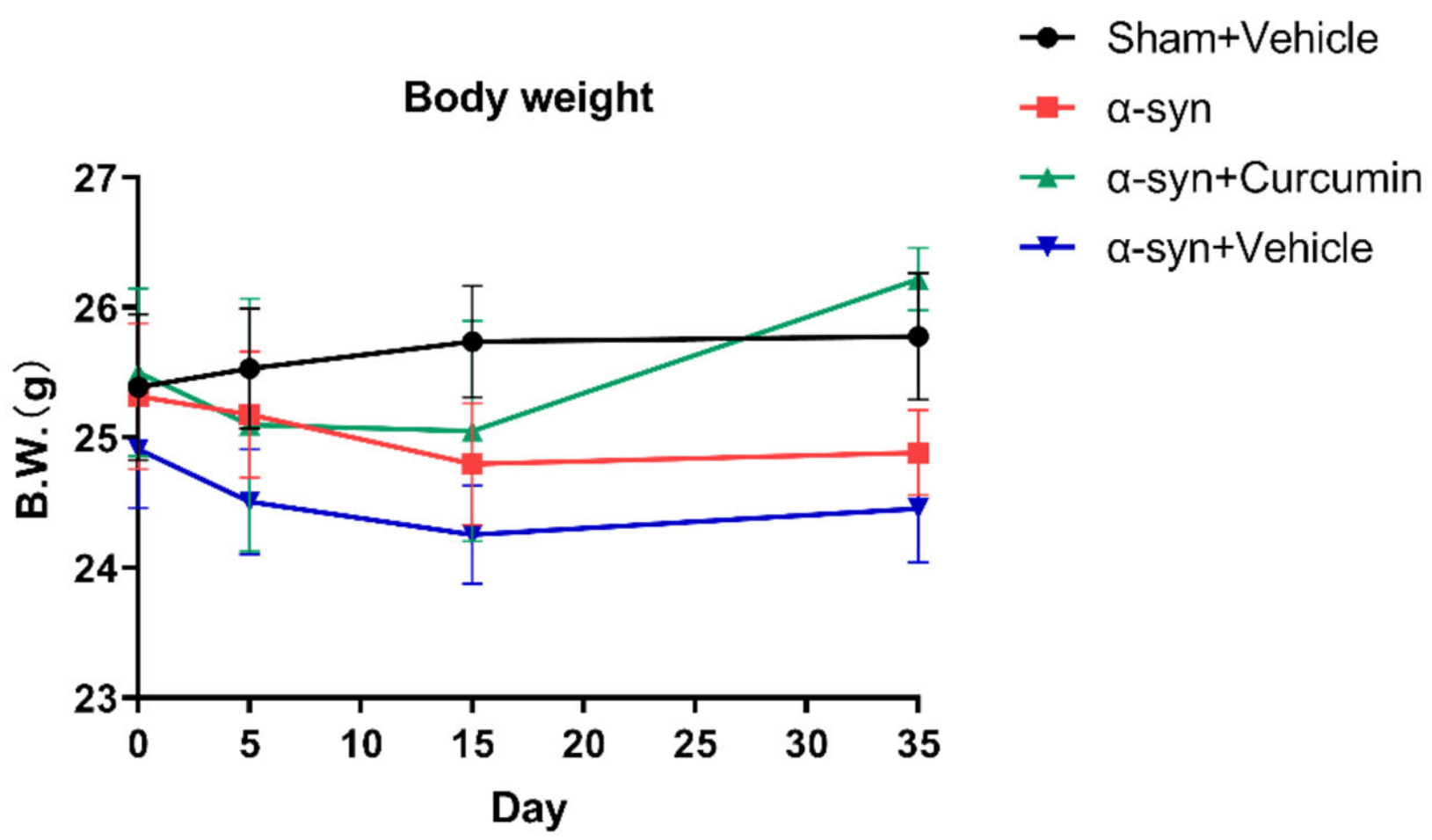

Supplementary Figure 5. **Body weight of mice in all groups.** Body weight of mice in all groups was evaluated during the whole experiment. n= 6 animals for each group**.**
